# Bio-Based Polyurethane Networks Containing Sunflower Oil Based Polyols

**DOI:** 10.3390/ijms25137300

**Published:** 2024-07-02

**Authors:** Katalin Czifrák, Csilla Lakatos, Csaba Cserháti, Gergő Vecsei, Miklós Zsuga, Sándor Kéki

**Affiliations:** 1 Department of Applied Chemistry, Faculty of Science and Technology, University of Debrecen, Egyetem tér 1, H-4032 Debrecen, Hungary; czifrak.katalin@science.unideb.hu (K.C.); lakatoscsilla@science.unideb.hu (C.L.); zsuga.miklos@science.unideb.hu (M.Z.); 2 Department of Solid State Physics, Faculty of Science and Technology, University of Debrecen, Bem tér 18/b, H-4026 Debrecen, Hungary; cserhati.csaba@science.unideb.hu (C.C.); vecsei.gergo@science.unideb.hu (G.V.)

**Keywords:** natural polymers, sunflower oil, glyceride mixture, polyurethane, crosslinking, biocompatible scaffold

## Abstract

This work focused on the preparation and investigation of polyurethane (SO-PU)-containing sunflower oil glycerides. By transesterification of sunflower oil with glycerol, we synthesized a glyceride mixture with an equilibrium composition, which was used as a new diol component in polyurethanes in addition to poly(ε-caprolactone)diol (PCLD2000). The structure of the glyceride mixture was characterized by physicochemical methods, matrix-assisted laser desorption/ionization time-of-flight mass spectrometry (MALDI-TOF MS), nuclear magnetic resonance spectroscopy (NMR), and size exclusion chromatography (SEC) measurements. The synthesis of polyurethanes was performed in two steps: first the prepolymer with the isocyanate end was synthesized, followed by crosslinking with an additional amount of diisocyanate. For the synthesis of the prepolymer, 4,4′-methylene diphenyl diisocyanate (MDI) or 1,6-hexamethylene diisocyanate (HDI) were used as isocyanate components, while the crosslinking was carried out using an additional amount of MDI or HDI. The obtained SO-PU flexible polymer films were characterized by attenuated total reflectance Fourier transform infrared spectroscopy (ATR-FTIR), differential scanning calorimetry (DSC), thermogravimetric analysis (TGA), and scanning electron microscopy (SEM). The so-obtained flexible SO-PU films were proved to be suitable for the preparation of potentially biocompatible and/or biodegradable scaffolds. In addition, the stress versus strain curves for the SO-PU polymers were interpreted in terms of a mechanical model, taking into account the yield and the strain hardening.

## 1. Introduction

Polyurethanes (PUs) are special representatives of polymers due to their versatile applications [1,2,3,4] and their freely varying structure. The scope of PUs’ applications can be widened by the use of building blocks of natural origins (e.g., modified vegetable oil-derived polyols) instead of the overwhelmingly petroleum-derived polyols [5,6].

In addition to being recyclable and non-toxic, vegetable oils can be functionalized using a number of processes [7,8,9]. Vegetable oils are usually built up from triglycerides of different fatty acids containing separated double bonds in the *cis* configuration [10], and they can be converted into polyols by transesterification [7], thiol-ene addition [11], epoxidation [9,12], or ozonolysis (followed by ring opening) [8].

It should also be noted that epoxidized vegetable oils are used as plasticizers, co-activators [13], and as substitutes for petroleum-based components in the rubber industry [14].

Among the vegetable oils, sunflower oil is a cheap and easily available oil that contains a high proportion of double bonds per triglyceride and consists mostly of C_18_ fatty acid chains. Its composition depends on the growing area [10]. The transformation of sunflower oil into polyols and their application in the synthesis of polyurethanes is the topic of several publications [8,15]. In addition, due to the presence of long-chain fatty acids as biocompatible/biodegradable building blocks, the oil may significantly influence the mechanical, thermal, and thermomechanical properties of the polymer [16,17].

Long-chain fatty acids can soften the resulting polymer, e.g., by increasing its flexibility and elasticity. However, the existence of the crosslinked structure can bring about the opposite effect due to the presence of a high number of branches and/or netpoints [18], i.e., the reactivity and functionality of the crosslinking agent significantly influence the number of branches and/or netpoints. As has been demonstrated, a number of multifunctional polyols or isocyanates can be used as crosslinkers in addition to urea allophanate or carbamate bonding [19,20].

Our main goal was the synthesis and investigation of a polyurethane-containing glyceride mixture formed from sunflower oil. In this scenario, polyurethane prepolymers with reactive isocyanate chain-ends were formed with polyol (glyceride mixture, GM) obtained from sunflower oil by transesterification and poly(ε-caprolactone)diol, followed by crosslinking with additional amounts of diisocyanate. In this work, we wanted to establish (i) what extent of the polyol produced from sunflower oil can be used as a compatibilizing agent, and (ii) how the presence of the glyceride mixture affects the mechanical and thermal properties of the polyurethane. (iii) Since polyurethanes can also be used as scaffolds in many fields in regenerative medicine [21], e.g., in orthopedics and dentistry [22,23], our further goal was to produce a potentially biocompatible and biodegradable scaffold.

Since the raw materials used for the production of polymers are biocompatible, the polymers produced from them can presumably be endowed with these properties [22,23,24].

## 2. Results and Discussion

### 2.1. Synthesis of Mixtures of Glycerides (MGs) from Sunflower Oil

**Figure 1:**
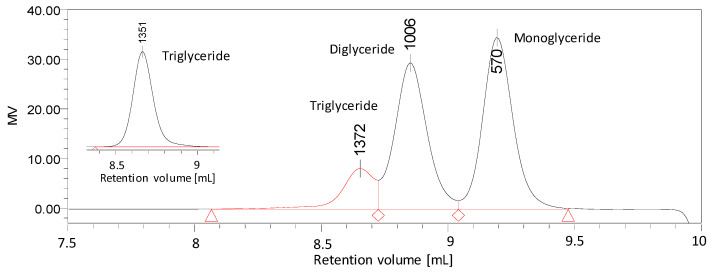
SEC chromatograms of sunflower oil and glyceride mixture (the values at the peaks refer to the peak molecular weights (M_p_)). The inset shows the SEC trace of the starting triglyceride.

**Figure 2:**
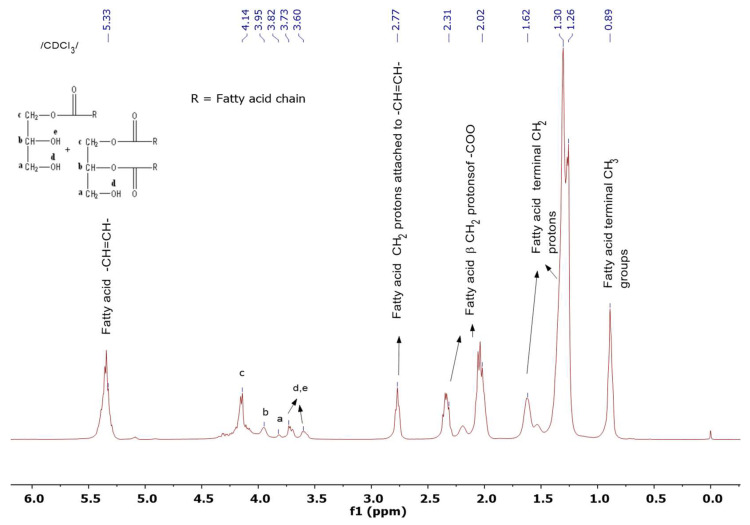
^1^H-NMR spectrum of glyceride mixture.

Sunflower oil alone, as a fatty acid triglyceride, does not contain reactive functional groups (e.g., hydroxyl groups). Therefore, the sunflower oil was transesterified with twice the amount of glycerine, resulting in an equilibrium mixture of glycerides. The reactions were carried out under an Ar atmosphere to avoid oxidation processes. The composition of the glyceride mixtures was investigated by SEC (Figure 1), physicochemical methods (Table 1), NMR (Figure 2 and Figure S1), and MALDI-TOF MS measurements (Figures S2–S4).

The SEC measurements confirmed that the transesterification of sunflower oil with glycerine resulted in an equilibrium mixture. Based on the analysis, the mixture contains 42% monoglyceride, 38% diglyceride, and 10% initial triglyceride.

The results of the physicochemical analysis of the sunflower oil and glyceride mixture are summarized in Table 1. As seen in Table 1, in addition to the increased acid number and hydroxyl number, the decreasing iodine number also reflects the success of the transesterification reaction.

The structure of the glyceride mixture was confirmed by ^1^H- and ^13^C-NMR measurements (Figure 2 and Figure S1). The assignment of the characteristic ^1^H-NMR peaks can be found in the Table 2.

The MALDI-TOF MS spectra of the mixture were also recorded, which were found to be consistent with the results of the SEC measurements (Figures S2–S4). In the MALDI-TOF MS spectrum of the sunflower oil, the ion detected with high intensity at *m/z* 903.888 belongs to the triglyceride, while the ions appearing with lower intensity at *m/z* 603.924 and *m/z* 427.780 correspond to the di- and monoglycerides, respectively.

### 2.2. Synthesis of SO-PUs

**Scheme 1:**
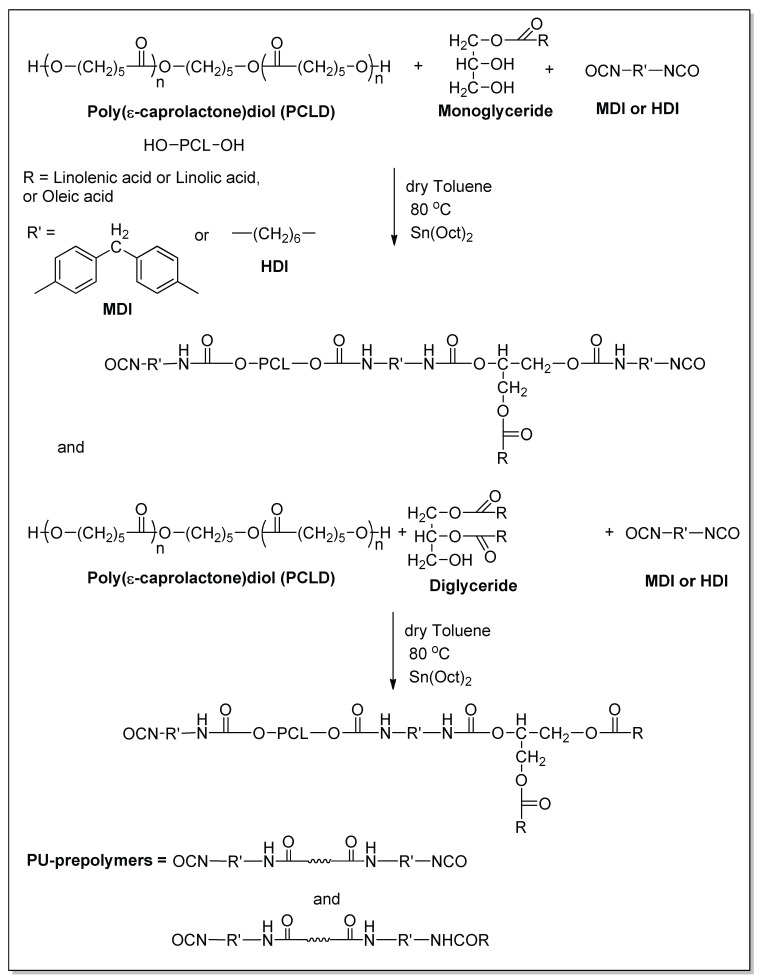
Reaction of PCLD2000 and glyceride mixture with diisocyanates. Synthesis of SO-PU prepolymers.

The syntheses of polyurethanes (SO-PUs) containing the poly(ε-caprolactone)diol (PCLD2000) and glyceride mixture (GM) were carried out by a two-step procedure, where the prepolymerization step was followed by crosslinking the polymer chains with an additional amount of diisocyanates. As a first approach, the prepolymer was prepared by adding the glyceride mixture to the reaction mixture after the reaction of PCLD2000 with diisocyanate was complete, then this was followed by crosslinking with MDI or HDI. However, this synthetic approach did not result in uniform films. Moreover, performing the reaction of glyceride, PCLD2000, and the diisocyanates in one step before the crosslinking step yielded uniform elastic films (Scheme 1).

During the prepolymerization the amounts of diols (PCLD2000 and GM) were kept constant, resulting mostly in NCO-terminated chains. The compositions of the reaction mixtures are listed in Table 10. The scaffold was successfully produced from sample SO-PU3, as described later.

**Scheme 2:**
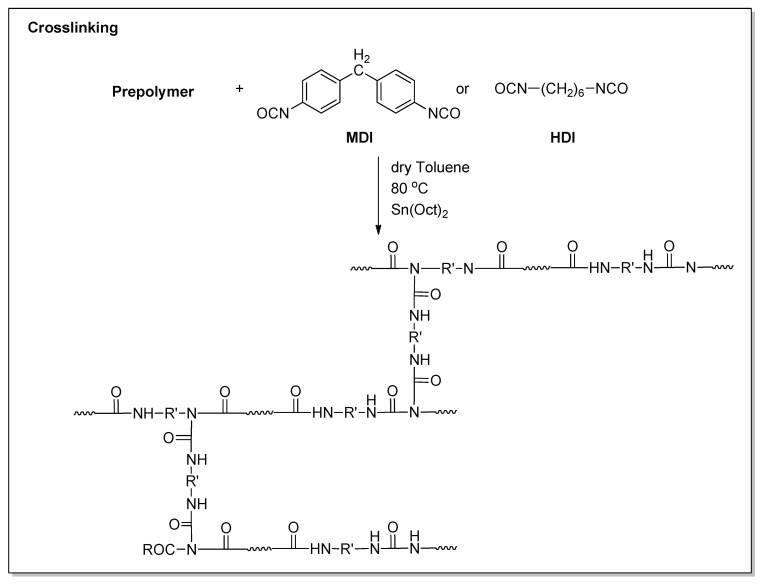
Crosslinking of SO-PUs.

The MDI or HDI forms allophanate bonds between the polymer chains to obtain a crosslinked structure, as shown in Scheme 2. The syntheses were performed in toluene in the presence of a tin-octoate catalyst. It was expected that the glyceride component could affect the thermal and the mechanical properties of the obtained polyurethanes.

### 2.3. Infrared Spectroscopy

**Figure 3:**
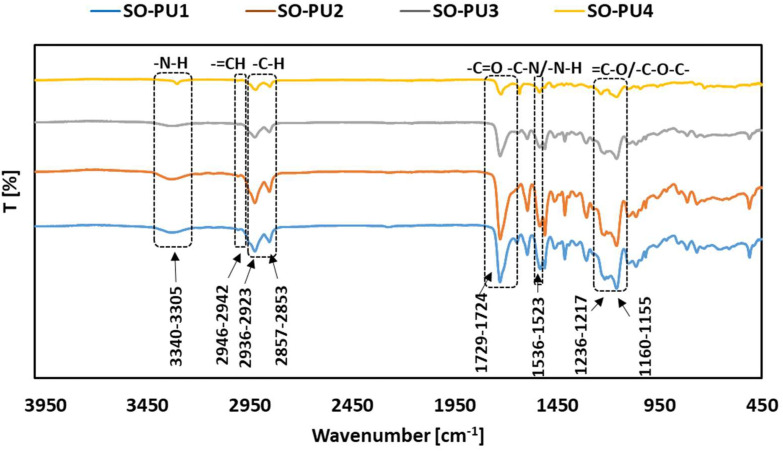
Infrared spectra of the synthesized samples SO-PU 1–4.

In order to verify the chemical structures of the polymers formed, SO-PU samples were investigated by IR (Figure 3). The broad absorption band between 3340 and 3305 cm^−1^ belongs to the urethane -NH stretching bands. The =C-H band of SO appears with low intensity between 2946 and 2942 cm^−1^ [25]. The -CH_2_ vibrations appearing around 2940 and 2860 cm^−1^ indicate the presence of a large number of CH_2_ groups in the PCLD2000 and oil units of SO-PUs. Importantly, the lack of an absorption band around 2230 cm^−1^ refers to the complete transformation of the NCO functional groups of the PU-prepolymers. The -C=O band appears between 1729 and 1724 cm^−1^ and the band between 1536 and 1523 cm^−1^ belongs to the –NH bending vibration. Furthermore, the vibrations of =C-O and –C–O–C– bonds occur between 1236 and 1217 and 1160 and 1155 cm^−1^.

### 2.4. Swelling Experiments

To obtain information about the formed network, swelling experiments were carried out using toluene. In this study, the swelling degree (Q), values of the gel content (G), and crosslink densities were determined (Table 3). From the data in Table 3, it can be surmised that the values of the swelling degree vary between 1.5 and 15.7, the gel contents of the SO-PU samples span from 89.6% to 97.1%, meanwhile the crosslink densities obtained were in the order of between 10^−3^ mol/cm^3^ and 10^−5^ mol/cm^3^.

The obtained results confirm the formation of crosslinked structures. Interestingly, for sample SO-PU1, a small degree of crosslinking was observed in spite of the fact that no additional diisocyanate (i.e., crosslinking agent) was used for crosslinking. This finding indicates that in the absence of further crosslinking, only a very loose network can form by the reaction of the chains with the unreacted isocyanate groups. Furthermore, in line with the results of the tensile test (see Table 4), SO-PU2 has the highest crosslink density.

### 2.5. Tensile Test

**Figure 4:**
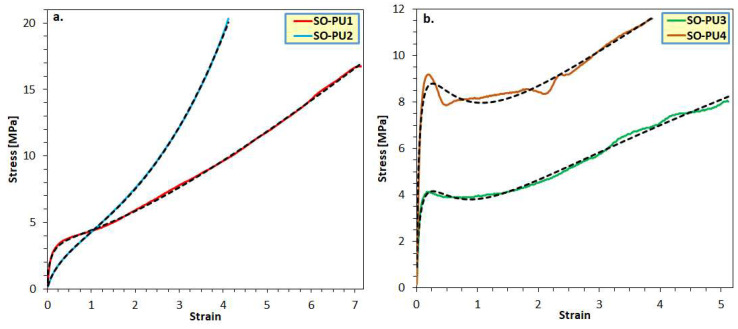
(**a**) Stress–strain curves for SO-PUs 1–2 and (**b**) SO-PUs 3–4. The dotted (black) curves represent the values fitted by Equation (1).

It can be concluded from the results of the tensile tests (Figure 4a,b, and Table 4) that there is a considerable change in the elongation at break, which decreases with the formation of networks with higher crosslink densities, as compared to the sample SO-PU1. This finding is consistent with the stiffening effect of the crosslinked structure. Consistently, the lowest elongation at break value was found for sample SO-PU2, for which the highest crosslink density was determined (see Table 3). However, there is no straightforward correlation between the elongation at break values and the types of crosslinker. The highest tensile strength value was found for SO-PU2 (18.1 MPa), while in the case of samples SO-PU1, SO-PU3, and SO-PU4 it varies between 8 MPa and 16 MPa. The elastic modulus values are higher for samples containing HDI in the main chain or as a crosslinker (SO-PU3 and SO-PU4, 81.5 and 160.8 MPa, respectively) (Table 4).

Furthermore, as expected, the presence of an almost fully amorphous structure gives higher elasticity to the samples (Table 4). Furthermore, as seen in Figure 4a,b, strain-hardening occurs for each sample, although at different strains. Stress versus strain curves with strain-hardening effects similar to those presented in Figure 4a have been successfully described by our mechanical model involving the extended Standard Linear Solid (SLS) model with a strain-dependent modulus (E-SLS) [26]. However, the most striking difference between the σ–ε curves is the presence of well-developed yield points in the cases of samples SO-PU3 and SO-PU4 (see Figure 4b). Although the E-SLS proved to be an appropriate model for describing the σ–ε properties for the elastic materials with strain-hardening, it is not suitable for rendering the mechanical properties of polymers with yield points such as those shown in Figure 4b. In an attempt to describe such complex σ–ε curves, we applied a more general expression for this purpose (Equation (1)). Equation (1), which is also applied, is suitable for interpreting the stress–elongation curves of materials where the material goes through two or more phases during the test, thus experiencing more instability phenomena [27].

(1) $$\sigma =\sum_{\iota =1}^{p}C_{i}\varepsilon^{n_{i}}\exp\left[-{\sum_{j=1}^{q}\left(\frac{\varepsilon -\varepsilon_{aj}}{\varepsilon_{0j}}\right)}^{m_{j}}\right]$$

where *C_i_*, *n_i_*, *ε_aj_*, *ε_*0*j_*, and *m_j_* are the parameters for the given material. More specifically, *n_i_* is the exponent for the actual relative strain (*ε*), *m_j_* is the Weibull parameter, while *ε_a_*_j_ and *ε*_0_*^j^* stand for the critical relative strain and C_i_ is the weighting (scaling) stress factor. In our case, we treated them as fitting parameters, p and q are natural numbers representing the number of phases and the transitions (or instability processes).

The stress–strain relationship in Equation (1) describes materials with multiple nonlinear elastic properties due to the presence of several phases, including also strain-hardening characteristics. Our SO-PU samples, as the data in Table 5 show, consist of two phases, namely, crystalline and amorphous phases in varying ratios. In the case of samples SO-PU1 and SO-PU2 the crystalline part is small (2% and 8%, respectively), while for SO-PU3 and SO-PU4 it is considerable higher (close to 30%). Therefore, there are two potential phases present (crystalline and amorphous) in the samples, so Equation (1) consists of two members and it can now be written as Equation (2):

(2) $$\sigma =C_{1}\varepsilon^{n_{1}}\exp\left[-{\left(\frac{\varepsilon -\varepsilon_{a1}}{\varepsilon_{01}}\right)}^{m_{1}}\right]+C_{2}\varepsilon^{n_{2}}\exp\left[-{\left(\frac{\varepsilon -\varepsilon_{a2}}{\varepsilon_{02}}\right)}^{m_{2}}\right]$$

Equation (2) was fitted to the σ versus ε curves trying to estimate the corresponding parameters. The preset and the fitted parameters are compiled in Table 5. As seen in Figure 4a,b, Equation (2). describes the σ–ε behaviors appropriately.

### 2.6. Thermal Properties

**Figure 5:**
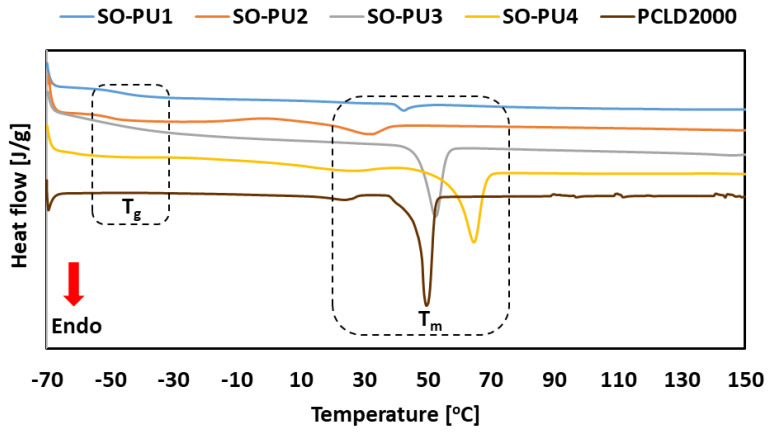
DSC traces of PCLD2000 [28] and SO-PUs 1–4 from first heating cycle.

The thermal behavior of the SO-PUs was investigated by DSC and TGA measurements. Figure 5 illustrates the DSC curves, while Table 6 contains the glass transition (T_g_) and melting temperature (T_m_), melting enthalpy (ΔH_m_), and the calculated degree of crystallinity (DC_r_). The T_g_ values vary between −50 °C and −40 °C in SO-PUs 1–3, but are not detected for SO-PU4. The crystallinity of the SO-PU samples is due to the presence of crystalline PCLD2000 domains. The degree of crystallinity was well below 60% in the case of all SO-PU samples. These fractions of crystallinity for SO-PU samples were considerably lower than that of the pure PCLD2000, indicating the predominant role of the crosslinking (i.e., crosslinking density) and softening ability of GM on the crystallization processes.

Indeed, the presence of GM reduces the crystallization tendency of the caprolactone unit, and thus, gives a mainly amorphous character to the samples. This effect is even more clear in the case of samples containing MDI in the main chain (see SO-PUs 1–3). Sample SO-PU1, most probably due to the softening effect of GM, and sample SO-PU2, because of the high crosslink density, show only a very low degree of crystallinity. On the contrary, however, samples SO-PU3 and SO-PU4, with moderate crosslink densities, reveal a higher degree of crystallinity compared to samples SO-PU1 and SO-PU2 (Table 6). This finding may be attributed to the confinement effect on the crosslinks in PCLD2000, meaning they cannot to arrange themselves into crystalline domains (a high crosslink density may cause the opposite effect). Interestingly, it can also be seen from the results of the DSC investigations that the crosslinks inhibit the melting of samples SO-PU3 and SO-PU4, as their T_m_ values are higher than that of PCLD2000 (Table 6). Furthermore, the T_m_ value of the slightly crosslinked sample (SO-PU1) (42 °C) is very similar to that of the native PCLD2000 (47 °C), while the T_m_ of the moderately crosslinked samples SO-PU3 and SOPU4 are significantly higher, 52 °C and 64 °C, respectively.

**Figure 6:**
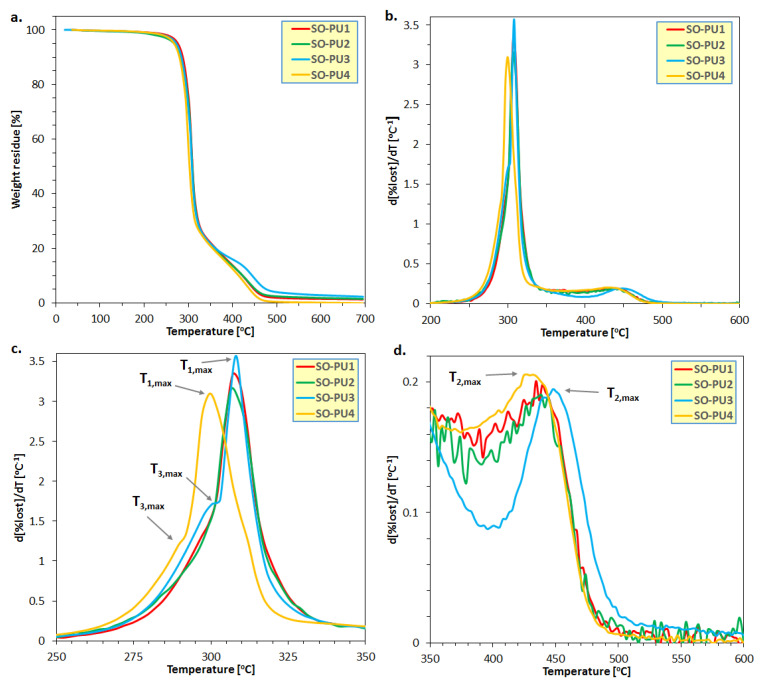
TGA curves for SO-PUs 1–4: (**a**) Weight residue versus temperature curves. (**b**) Relative weight loss rate (d[%lost]/dT) as a function of the temperature. (**c**) d[%lost]/dT versus T curves in the temperature range of 250–350 °C. (**d**) d[%lost]/dT versus T curves in the temperature range of 350–600 °C.

The thermal stabilities of the samples were also studied by TGA measurement (Figure 6).

As seen in Figure 6a, each sample degrades by a two-step process at around 300 °C and 430 °C and the thermal stabilities of these samples are very close to each other. This latter finding suggests that the thermal stabilities for these samples are mainly determined by the presence of ester and urethane bonds. The decomposition temperatures at 5%, 10%, and 50% weight loss and at the end-set temperature are summarized in Table 7. Indeed, the data in Table 7 reveal similar thermal stability for all the four samples. However, in order to obtain a deeper insight into the degradation processes, the relative weight loss rates as a function of temperature were also plotted and are shown in Figure 6b–d. In addition, the values of T_max_, i.e., temperature at which the maxima on the d[%lost]/dT versus temperature curves occur are also gathered in Table 8. As seen from the data in Table 8, two T_max_ values (T_1,max_ and T_2,max_) can be deduced for samples SO-PU1 and SO-PU2, whereas for samples SO-PU3 and SO-PU4 there are additional T_3,max_ values appearing as a shoulder before T_1,max_. The first main degradation step, occurring at ca. 310 °C, can be attributed to the decomposition of PCLD2000, which may overlap with the degradation of the urethane linkages. The second stage, at around 430 °C, corresponds to the degradation processes of the polyol mixture (GM). The additional characteristic maxima (T_3,max_) appearing as shoulders at around 300 °C, are most likely due to the decomposition of the allophanate linkages.

### 2.7. Morphology

**Figure 7:**
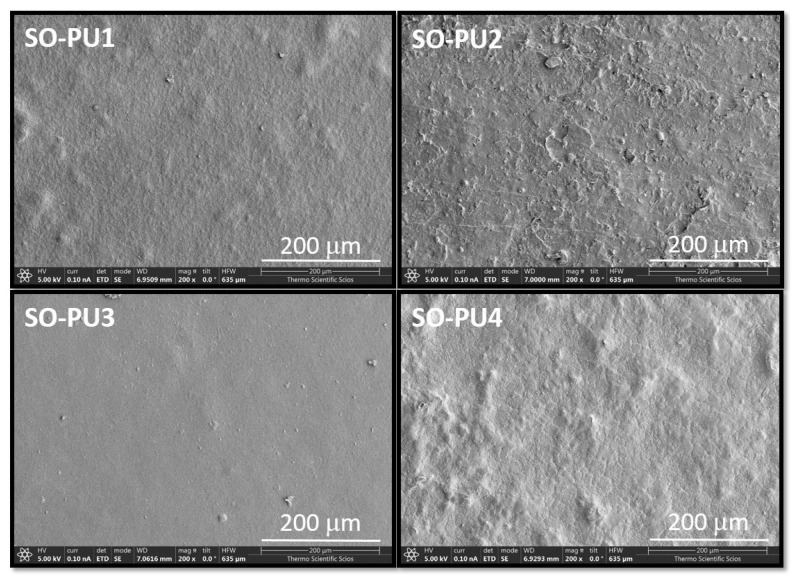
SEM images of SO-PUs 1–4.

To obtain a deeper insight into the morphology and the compatibility effect of the glyceride mixture, SEM images were taken of the samples (Figure 7). As seen in Figure 7, while the surface of sample SO-PU3 seems to be uniform, those of others crosslinked with MDI (SO-PUs 2 and 4) are somewhat uneven and bumpy. From the morphological differences, it can be established that an almost completely smooth surface can be obtained in the case of crosslinking by HDI (SO-PU3), indicating better compatibility with the oil-based glyceride mixture. However, the use of MDI as a crosslinker results in a higher crosslink density at the expense of compatibility (see SO-PUs 2 and 4).

### 2.8. SEM Investigation of Scaffold from SO-PU3

**Figure 8:**
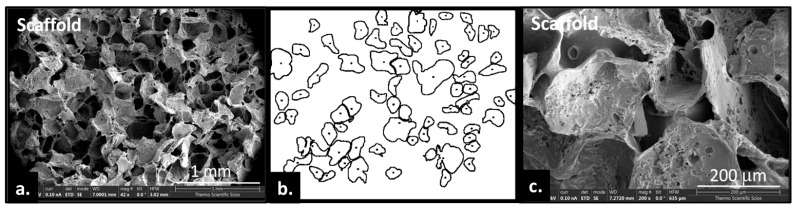
(**a**) SEM images of SO-PU3 scaffold; (**b**) the outlines of the pores examined; and (**c**) zoomed SEM image of SO-PU3.

The scaffold prepared from SO-PU3 according to the method detailed in the Section 2.4. was also examined using SEM. The SEM images of the scaffold are shown in Figure 8. The SEM images reveal the formation of an open-cell structure. The open-cell structure enables the use of the scaffold as a potential soft tissue substitute. The pore sizes, major and minor ellipses, and Feret diameters of the scaffold were determined from the SEM images with the ImageJ software [30]. The results of the SEM image evaluation are summarized in Table 9. As seen from the data in Table 9, the major and minor diameters were 230 μm and 140 μm, respectively, showing an uneven cell diameter and shape of the pores. Furthermore, the distribution of the pore size varies also in a relatively large range, providing various effective binding sites for microorganisms.

## 3. Materials and Methods

### 3.1. Materials

For the experiments, commercially available Sunflower oil refined after pressing and/or extraction was used (distributor: Lidl Magyarország Ltd., Budapest, Hungary). Poly(ε-caprolactone)diol (PCLD2000) (M_n_ = 2 kg/mol), 1,6-Hexamethylene diisocyanate (HDI), 4,4’-Methylene diphenyl diisocyanate (MDI), and catalyst Tin(II) 2-ethylhexanoate were purchased from Sigma-Aldrich Chemical Co. (Darmstadt, Germany). Toluene (analytical grade, stored on sodium wire) from Molar Chemicals Ltd. (Halásztelek, Hungary) and Dimethyl-sulfoxide (99.9%, DMSO, stored on molecular sieve) from Sigma-Aldrich (Darmstadt, Germany) were used as received.

### 3.2. Transesterification of Sunflower Oil with Glycerine

Transesterification of sunflower oil was carried out similarly to as described in Ref [31]. In a three-necked flask, under an argon atmosphere, 5 g of sunflower oil and 10 g of glycerine (mass ratio of sunflower oil to glycerine was 1:2) were heated at 170 °C under an argon atmosphere for 6 h in the presence of 0.05 wt% of calcium oxide. The glycerides were obtained by dissolving the reaction mixture in n-hexane and washed with water 5 times. After drying over magnesium sulfate, the n-hexane was removed by evaporation to give 3.7 g of thick yellow oil. The glyceride mixture (GM) was characterized with physicochemical methods [32], NMR spectroscopy (^1^H- and ^13^C-NMR), MALDI-TOF MS, and SEC measurements.

### 3.3. Synthesis of SO-PUs

Diols (PCLD2000 and GM) were dissolved in dry toluene (30 mL), in a three-necked flask equipped with an Ar gas in–outlet and a condenser, under stirring at 80 °C. After dissolution of the solid particles, diisocyanate MDI or HDI and tin(II) 2-ethylhexanoate as a catalyst (2 mol%, 0.032 g) were added to the solution and stirred for two hours at 80 °C. Crosslinking was carried out by adding a further amount of MDI or HDI to the reaction mixture and stirring for an additional two hours at 80 °C. The viscous product was poured into a Teflon^®^ vessel and left for 72 h in air to dry. The obtained elastic polymer films were characterized. The compositions of the reaction mixtures are compiled in Table 10.

### 3.4. Preparation of Scaffold from SO-PU3 by Salt Leaching

The SO-PU3 sample was produced in the same way as described previously (see Section 2.3). The scaffold was prepared similarly to those reported in the literature [33,34]. To the toluene solution poured into the pan, 65 g of ground and graded NaCl with a diameter of 200 µm to 250 µm was added and mixed. The mixture was then left to dry at room temperature for 3 days. The application of this condition resulted in a ~2 mm thick film, from which the salt was washed by soaking in pure water. The water was removed in an oven at 40 °C overnight. This resulted in a flexible open-cell sponge-like material.

### 3.5. Characterization

#### 3.5.1. Characterizations of Sunflower Oil and GM

##### Physicochemical Characterization

The acid values were determined according to the ISO 660:2009 [35] official method and the iodine values were calculated from the fatty acid composition using the method described by the ISO 3961:2018 [36] standard. Hydroxyl values were obtained using the international standard ISO 4629-2:2016 [37] official method.

##### Matrix-Assisted Laser Desorption/Ionization Time-of-Flight Mass Spectrometry (MALDI-TOF MS)

The matrix-assisted laser desorption/ionization time-of-flight mass spectrometry (MALDI-TOF MS) spectra were recorded with a Bruker Autoflex Speed mass spectrometer equipped with a time-of-flight/time-of-flight (TOF/TOF) mass analyzer (Bruker Daltoniks, Bremen, Germany). Ions were detected in the positive ion mode in the reflectron and in the linear mode. A solid-phase laser (355 nm, ≥100 µJ/pulse) operating at 500 Hz was applied to produce laser desorption and 5000 shots were summed. The MS spectra were externally calibrated with a polyethylene glycol standard (M_n_ = 1540 g/mol). The samples for MS measurement were prepared with a 2,5-dihydroxy benzoic acid (DHB) matrix dissolved in THF at a concentration of 20 mg/mL. The samples and the sodium trifluoroacetate used as an ionizing agent were also dissolved in THF at concentrations of 10 mg/mL and 5 mg/mL, respectively. The applied mixing ratio was 10/2/1 (*V*/*V*) (matrix/sample/cationizing agent). A volume of 0.25 µL of the so-obtained solution was deposited onto a metal sample plate and allowed to dry in air.

##### NMR Spectroscopy

^1^H- and ^13^C-NMR spectra were recorded with a Bruker AM 360 (360/90 MHz for ^1^H/^13^C) spectrometer (Bruker, Karlsruhe, Germany). Deuterated chloroform was used as the solvent and Me_4_Si was used as the standard.

##### Size-Exclusion Chromatography (SEC)

SEC chromatograms were recorded in dimethylformamide (DMF) at a flow rate of 0.5 mL/min with a Waters chromatograph equipped with four gel columns (4.6 × 300 mm, 5 µm Styragel columns: HR 0.5, 1, 2 and 4), a Waters Alliance e2695 HPLC pump, and with a Waters 2414 refractive index detector (Waters Corp., Milford, MA, USA). The SEC was calibrated with polystyrene standards.

#### 3.5.2. Characterization of SO-PU 1–4 Samples

##### Attenuated Total Reflectance Fourier Transform Infrared (ATR-FTIR)

Attenuated total reflectance Fourier transform infrared spectroscopy (AT-FTIR) measurements were carried out with a Perkin Elmer Instrument (Waltham, MA, USA) Spectrum Two FTIR spectrometer. The Spectrum Two FTIR spectrometer was equipped with a diamond Universal ATR Sampling Accessory. Eight scans were taken from specimens with a ~0.5 mm thickness. Data collection was carried out in the spectral range of 8300–350 cm^−1^, with a best resolution of 0.5 cm. The obtained IR spectra were evaluated by the Spectrum ES 5.0 program.

##### Scanning Electron Microscopy (SEM)

The SEM pictures were taken from the surface of the PUs with a Thermo Fisher Scientific Scios 2 dual-beam scanning electron microscope (FIB-SEM). The images were taken using a 2 kV acceleration voltage, 25 pA beam current, and short dwell time (100 ns) in secondary electron (SE) mode after covering the samples with a 30 nm conductive gold layer.

##### Swelling Experiments

The parameters measured were the degree of swelling (Q), gel content (G), and crosslink density (ν_e_). The samples (dimensions: 10 mm × 10 mm × ~0.5 mm) were swollen in toluene (20 mL) at 20 °C (294 K) in a closed bottle for 48 h. The degree of swelling (Q), the gel content (G), and the crosslink density were calculated by Equations (3)–(5) [38].

(3) $$Q=1+\frac{\rho_{s}}{\rho_{p}}\cdot (\frac{m_{2}}{m_{3}}-1)$$

(4) $$G(\%)=\frac{m_{3}}{m_{1}}\cdot \;100$$

where ρ_s_ and ρ_p_ are the densities of the solvent (toluene, ρ: 0.8669 g/cm^3^) and the PU polymer, respectively.

The crosslink density (ν_e_) was calculated based on the swelling results using the Flory–Rehner Equation [39]:

(5) $$\nu_{e}=\frac{-\left[\ln(1-v_{1})+v_{1}+\chi \cdot v_{1}^{2}\right]}{V_{ms}\cdot (v_{1}^{1/3}-\frac{v_{1}}{2})}$$

where ν_e_ = crosslink density, V_ms_ is the molar volume of the solvent (1.06 × 10^−4^ m^3^/mol), χ is the polymer–solvent interaction parameter (calculated to be 0.230 at 294 K), and ρ_p_ is the density of the polymer [40].

##### Mechanical Tests

Uniaxial tensile measurements were carried out by using a computer-controlled INSTRON 3366 universal testing machine (INSTRON, Norwood, MA, USA). According to the ASTM D882-12 standard, 5 specimens were cut from each sample with ~0.5 mm thickness (clamped length: 60 mm) and tested with a crosshead speed of 50 mm/min after conditioning. The E-moduli (Young moduli), stress, and strain values were obtained from the stress–strain curves.

##### Differential Scanning Calorimetry (DSC)

The DSC tests were carried out with a DSC Q2000 power condensation instrument (TA Instruments, New Castle, DE, USA). During the measurements heat/cool/heat cycles were used. During the heating cycle the temperature was raised from −70 °C to 220 °C at a 10 °C/min heating rate. The sample cooled down from 220 °C to −70 °C in the cooling cycle with the same heating rate. The crystallinity value of PCLD2000 segment was calculated by Equation [41]:

(6) $$C_{r}=\frac{\Delta H_{m}}{\chi_{A}\;\cdot \;\Delta H_{m}^{0}}\cdot \;100\;\%$$

where ΔH_m_ is the heat of fusion of the measured PU derivatives, and $\chi_{A}$ is the weight fraction of PCLD2000. $\Delta H_{m}^{0}$ is the heat fusion of the pure 100% crystalline PCLD2000 [42].

##### Thermogravimetric Analysis (TGA) 

The TGA thermograms were recorded with a TGA Q500 instrument (TA Instruments, New Castle, DE, USA) with a nitrogen flow rate of 30 mL/min at a heating rate of 10 °C/min. During the heating the temperature was raised from 40 °C to 800 °C.

## 4. Conclusions

The main goal of our work was the synthesis and characterization of polyurethanes containing a glyceride mixture (SO-PUs) in order to study the effect of a polyol mixture originating from native sunflower oil. For the synthesis of the SO-PU samples, MDI or HDI and a sunflower oil-based polyol (glyceride mixture) was used in addition to ε-caprolactonediol (PCLD2000). The crosslinking between the polyurethane chains was carried out by adding a further amount of MDI or HDI to the reaction mixture. The properties of the SO-PUs were investigated systematically by ATR-FTIR, swelling, mechanical, and thermal methods. Of the investigated samples, the weakly (SO-PU1) and highly crosslinked (SO-PU2) samples showed low crystallinity, whereas for the moderately crosslinked samples, SO-PU3 and SO-PU4, higher crystalline fractions were found. Based on the SEM images, it was pointed out that the more flexible HDI shows greater compatibility with the polyols derived from sunflower oil. Furthermore, we also demonstrated that a flexible tissue scaffold can be produced from the SO-PUs polyurethanes. In addition, for the interpretation and description of the σ versus ε curves, a mechanical model consisting of two exponential terms, taking into account the yield and strain-hardening effect, was also suggested. In the course of our work, we demonstrated that polyurethanes with good mechanical properties can be produced by using a polyol formed from sunflower oil, which may also be suitable for the production of tissue-substitute scaffold material.

## Figures and Tables

**Table 1:** The results of the physicochemical analysis of the refined sunflower oil and glyceride mixture.

Analysis	Sunflower Oil	Glyceride Mixture (GM)
Density (g/cm^3^)	0.87	0.95
M_n_ (g/mol)	1319.4	mono: 990.5
		di: 558.1
Acid value (mg KOH/g)	0.56	15.9
Iodine value (g I_2_/100 g)	131.5	59.5
Hydroxyl value (mg KOH/g)	2.8	222

**Table 2:** Assignment of ^1^H-NMR spectrum of glyceride mixture.

Protons	σ (ppm)/CDCl_3_/
Fatty acid -CH=CH-	5.39–5.29
CH_2_ glyceride	4.17–4.11
CH glyceride	3.97–3.93
CH glyceride	3.84–3.80
OH	3.73–3.69
CH_2_ in fatty acid chain attached to CH=CH-	2.79–2.75
CH_2_ of fatty acid chain in β position to COO-	2.33–2.29
CH_2_ of fatty acid chain in β position to COO-	2.04–2.00
Terminal CH_2_ of fatty acid chain	1.63–1.25
Terminal CH_3_ of fatty acid chain	0.91–0.88

**Table 3:** The density, swelling degree (Q), gel content (G), and crosslink density (ν_e_) in toluene at 294 K.

Sample Name	Density (g/cm^3^)	Q	G (%)	ν_e_ (mol/cm^3^)
SO-PU1	1.09	8.9	91.8	4.5 × 10^−5^
SO-PU2	1.10	1.5	97.1	3.2 × 10^−3^
SO-PU3	1.11	15.7	93.4	2.1 × 10^−4^
SO-PU4	1.01	2.6	89.6	9.2 × 10^−4^

**Table 4:** Tensile mechanical properties of SO-PUs 1–4. Designations: E is the elastic modulus (Young’s modulus), ε_R_ is the ultimate elongation, and σ_R_ is the stress at break.

Sample Name	E (MPa)	ε_R_ (%)	σ_R_ (MPa)
SO-PU1	12.3 ± 1	718.7 ± 63	15.9 ± 1
SO-PU2	8.4 ± 0.5	358.5 ± 36	18.1 ± 1.5
SO-PU3	81.5 ± 25	461.3 ± 36	7.7 ± 0.7
SO-PU4	160.8 ± 7	386.7 ± 38	11.5 ± 1.6

**Table 5:** The determined mechanical parameters for samples SO-PUs 1–4.

	Sample			
Parameter	SO-PU1	SO-PU2	SO-PU3	SO-PU4
C_1_ *	270.1	65.48	34.06	336.8
**n_1_**	1	1	0.7	0.9
**ε_a1_**	0	0	0	0
**ε_01_**	0.00359	0.00205	0.140690	0.02259
**m_1_**	0.27076	0.16619	0.635541	0.40360
C_2_ *	4.5785	0.84026	7.6754	12.619
**n_2_**	1.152	2.600	0.678074	0.65273
**ε_a2_**	0	0	0.001853	0.00049
**ε_02_**	0.6949	0.69494	0.34652	0.69565
**m_2_**	0	0	0.01437	0

* The unit for C_1_ and C_2_ is MPa, while the others are dimensionless parameters. Parameters n_1_ and ε_a1_ were preset to the given values shown in the table to obtain the best fit.

**Table 6:** Glass transition (T_g_) and melting temperature (T_m_), melting enthalpy (ΔH_m_), and degree of crystallinity (DC_r_) obtained from the first heating cycle of DSC measurements of PCLD2000 and SO-PUs 1–4.

Sample Name	T_g_ (°C)	T_m_ PCLD (°C)	ΔH_m_ (J/g)	DC_r_ (%)
PCLD2000	-	47	85.3	60
SO-PU1	−43	42	1.3	2
SO-PU2	−50	32	6.0	8
SO-PU3	−50	52	21.6	28
SO-PU4	-	64	23.4	29

**Table 7:** Results of thermogravimetric analysis (TGA) for PCLD2000 [29] and SO-PUs 1–4 conducted under nitrogen atmosphere. T_5%_ is decomposition temperature at 5% weight loss, T_10%_ decomposition represents the temperature at 10% weight loss, T_50%_ stands for the decomposition temperature at 50% weight loss, and the endset is the final temperature.

Sample Name	T_5%_ (°C)	T_10%_ (°C)	T_50%_ (°C)	T_endset_ (°C)
PCLD2000	302	318	367	463
SO-PU1	275	287	309	463
SO-PU2	275	284	308	473
SO-PU3	267	278	302	460
SO-PU4	253	267	300	449

**Table 8:** T_max_ values determined from the d[%lost]/dT versus temperature data.

Sample Name	T_1,max_ (°C)	T_2,max_ (°C)	T_3,max_ (°C)
SO-PU1	307	435	n.a.
SO-PU2	306	441	n.a
SO-PU3	308	448	301
SO-PU4	300	429	292

**Table 9:** Pore sizing analysis result of SO-PU3 scaffold.

Sample Name	SO-PU3 Scaffold
Count	68
Major diameter (µm)	230 ± 80
Minor diameter (µm)	140 ± 50
Ellipse angle	87 ± 56
Feret max (µm)	240 ± 90
Feret min (µm)	150 ± 60
Feret angle	96 ± 55

**Table 10:** Compositions of reaction mixtures. Soft segment (SS) and hard segment (HS) contents.

Sample Name	PCLD2000 (g)	GM (g)	MDI (g)	HDI (g)	Crosslinker		SS (%)	HS (%)
					MDI (g)	HDI (g)		
SO-PU1	4	1	1.8				73.5	26.5
SO-PU2	4	1	1.8		0.5		68.5	31.5
SO-PU3	4	1	1.8			0.3	70.4	29.6
SO-PU4	4	1		1.3	0.5		74.5	25.5

## Data Availability

Data are available from the corresponding author upon request.

## Supplementary Materials

The following supporting information can be downloaded at: https://www.mdpi.com/article/10.3390/ijms25137300/s1.
