# Supplementary material for: Bio-Based Polyurethane Networks Containing Sunflower Oil Based Polyols
Source: Int J Mol Sci. 2024 Jul 2;25(13):7300. doi: 10.3390/ijms25137300 (PMC11242490; doi:10.3390/ijms25137300)
Supplement: Supplementary file 1 [file ijms-25-07300-s001.zip › ijms-3049609-supplementary.pdf]

Bio-based Polyurethane Networks Containing Sunflower Oil Based Polyols

Katalin Czifrák[1], Csilla Lakatos[1], Csaba Cserháti[2], Gergő Vecsei[2], Miklós Zsuga[1] and Sándor Kéki[1]

[1] Department of Applied Chemistry, University of Debrecen, Egyetem tér 1, H-4032 Debrecen, Hungary;

[2] Department of Solid State Physics, University of Debrecen, Bem tér 18/b, H-4026 Debrecen, Hungary

*Correspondence: keki.sandor@science.unideb.hu; Tel.: +36-52-512-900 (ext. 22455)

Table of contents

Figure S1. ^{13}C-NMR spectrum of glyceride mixture. . 3.

Figure S2. MALDI-TOF MS spectrum of sunflower oil. 4.

Figure S3. MALDI-TOF MS spectrum I of glyceride mixture. 5.

Figure S4. MALDI-TOF MS spectrum II of glyceride mixture. 5.

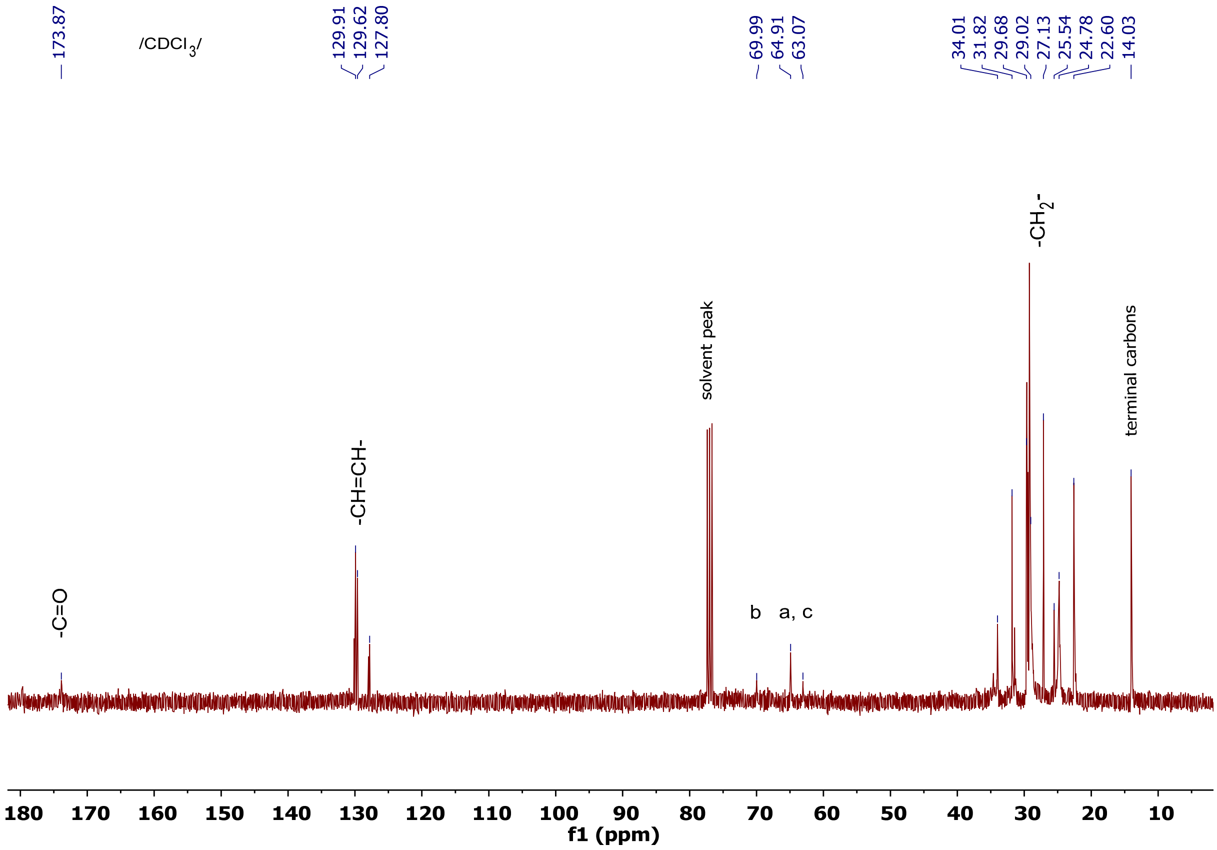

Figure S1. ^{13}C-NMR spectrum of glyceride mixture.

^{13}C-NMR ($CDCl_3$, 90 MHz,) σ ppm: 173.9 (CO), 130.0, 129.9, 129.6, 127.9, 127.8, (CH=CH), 70.0 (CH glyceride), 64.9 (CH_2 glyceride), 63.1 (CH_2 glyceride), 34.01, 31.8, 29.7, 29.0, 27.1, 25.6, 24.8, 22.6 (CH_2 of fatty acid chain), 14.0 (terminal CH_3 of fatty acid chain).

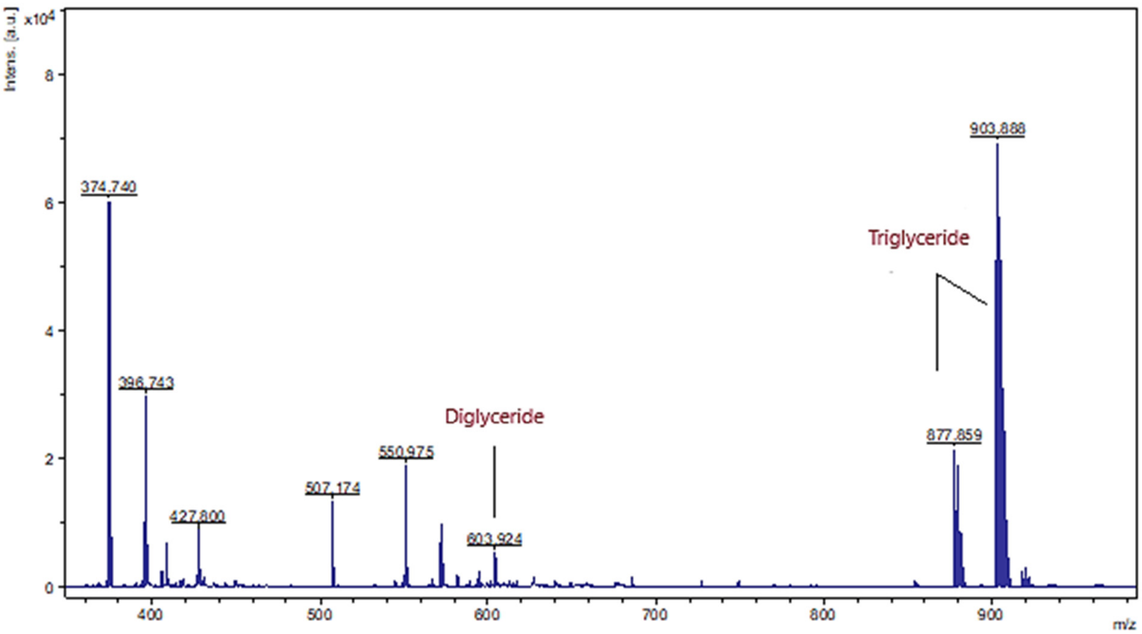

Figure S2. MALDI-TOF MS spectrum of Sunflower oil.

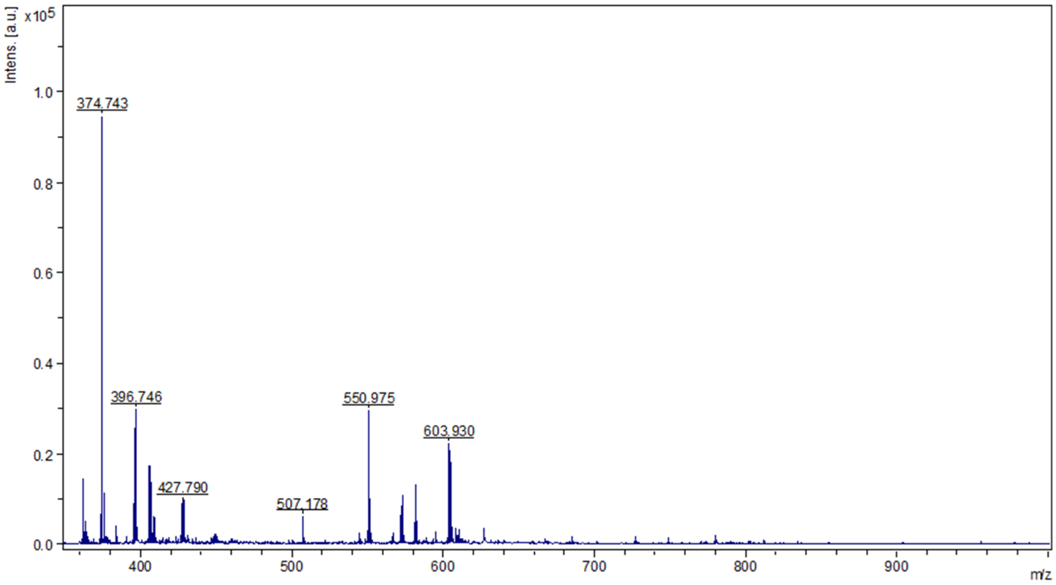

Figure S3. MALDI-TOF MS spectrum I of glyceride mixture.

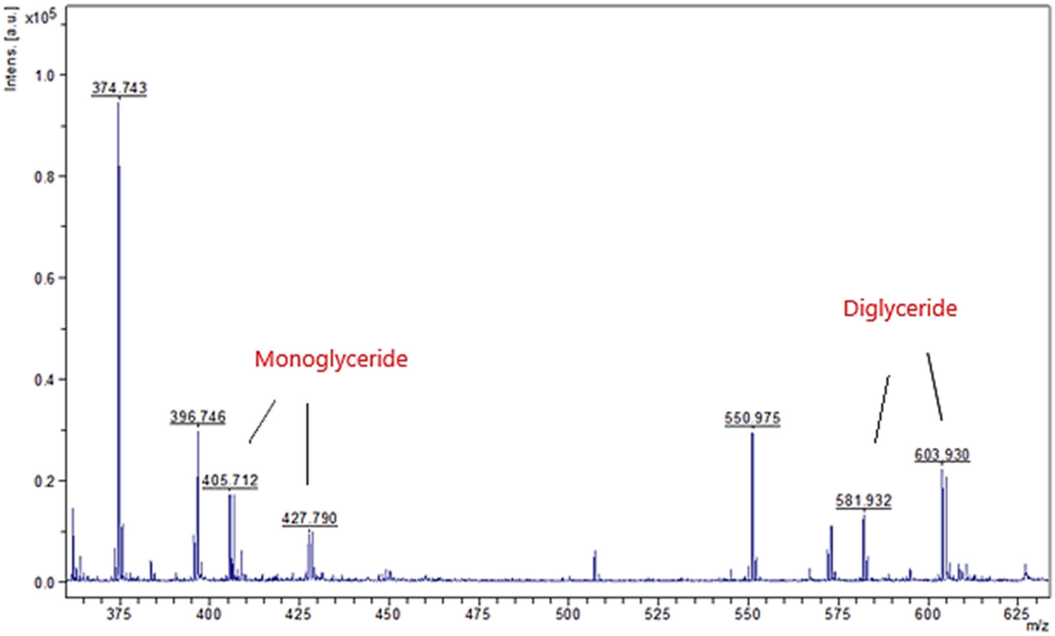

Figure S4. MALDI-TOF MS spectrum II of glyceride mixture.
